# A call for collaboration in surgical oncology

**DOI:** 10.1038/bjc.2011.446

**Published:** 2011-11-22

**Authors:** R W Glynn, K J Sweeney

**Affiliations:** 1 Discipline of Surgery, National University of Ireland Galway, Costello Road, Galway, Ireland

In their recent assessment of trends in cancer research since the establishment of the National Cancer Research Network in 2001, Stead *et al* (2011) reported significant improvement in both patient recruitment and study delivery over the intervening time period. This has been largely attributed to the development of the UK cancer centres, an initiative similar to those seen across the EU27 (Ringborg *et al*, 2008) and the United States (Nass *et al*, 2010). In the United States, the move towards service centralisation has, however, made it increasingly difficult for clinicians working outside designated departments to maintain a research output and bibliometric profile sufficient to facilitate future support and funding (Nass *et al*, 2010). This emerging organisational structure for cancer research holds particular significance for those working in surgical oncology.

In an enlightened editorial in the *Annals of Surgery*, Gerry O’Sullivan (O’Sullivan, 2010) pointed out that <1.5% of research funds in the United Kingdom are devoted to surgical research. Furthermore, there has been a 50% decline in the number of mid-career academic surgeons since 2000, and an overall decline of 20% in the total numbers of academic surgeons in the same period (Eckhouse and Sullivan, 2008). The heterogeneity in institutional support for academic cancer surgeons is of particular concern, and increased inter-institutional surgical cooperation must be supported, particularly between designated major cancer centres and peripheral institutions.

Indeed, scientific research in general is an increasingly collaborative, multi-disciplinary, multi-location, and multi-funded activity (Allen *et al*, 2009). Collaboration is associated with improved quality of research output as assessed using common bibliometric indicators (Trimble *et al*, 2009; Glynn *et al*, 2010b), although also facilitating the elucidation of clinically applicable advancements in care, as perhaps best demonstrated by the improved clinical outcomes associated with the Veterans Affairs’ National Surgical Quality Improvement Program in the United States (Khuri *et al*, 1998).

Despite the recognition of its importance, and the obvious benefits for participating institutions, it could be argued that surgeons have fallen behind when it comes to cooperative research. Of the 136 oral presentations given at the 2011 annual conference of the Society of Academic and Research Surgery, for example, just 12 (9%) had been the result of international collaboration. Furthermore, our analysis of authorship trends in the surgical literature demonstrated that, in 2008, only 44% of clinical trials published across the five highest impact factor surgical journals were multicentre; this compared with a figure of 68% across the top five medical journals (Glynn *et al*, 2010a). In addition, <4% of papers in leading surgical titles comprise the randomised controlled trials design (Smythe, 2010). Although this situation has been addressed in the United States with the establishment of the American College of Surgeons Oncology Group, which aims to engage surgeons as active participants in well-designed, multi-institutional trials (Wells, 2002), similar efforts are required elsewhere.

As funding follows quantifiable output, substantive surgical oncology research in the future may be beyond most individual surgical departments and may require a more networked approach (O’Sullivan, 2010). It is fortunate, then, that cancer has proven amenable to such an approach, as highlighted by the increases seen in multi-institutional research for individual cancers (Glynn *et al*, 2010b), and through the development of organisations including the European Organisation for Research and Treatment of Cancer, the European Cancer Organisation, and the Organisation of European Cancer Institutes.

These organisations have developed in tandem with the greater scientific, monetary and social ‘European Project’ over recent decades. Although the value of this project from a fiscal standpoint might currently be under scrutiny, the increased collaborative opportunities afforded by the development of the European Union have proved to be enormously beneficial to scientific endeavours, as exemplified by the success associated with institutions including the European Molecular Biology Laboratory and the European Organisation for Nuclear Research (CERN). Of relevance to the issues under discussion here, the European Strategy Forum on Research Infrastructures, launched in 2002, has led to the establishment of the European Clinical Research Infrastructure Network, the European Advanced Translational Research Infrastructure in Medicine (EATRIS), and eight other research infrastructures related to the biological and medical sciences (Castleton *et al*, 2011).

The challenge for those working in surgical oncology is to recognise and avail the opportunities afforded by the advent of these and similar networks in facilitating the development of collaborative research projects. These actions could provide the impetus for the production of a body of high quality, clinically applicable, research yield, with the associated benefits of improving the bibliometric profile of individual units, thereby advancing their case for future research funding and support. In an era when we compete not alone against our medical and biomedical colleagues for this support, but increasingly against unrelated research disciplines, including agriculture, climate change, and energy (O’Sullivan, 2010), those involved in surgical research must seek to improve through collaboration or risk being caught in a downward spiral of deteriorating research output and consequential loss of support for future initiatives.
